# Sawyer’s Forceps

**Published:** 1877-07-20

**Authors:** 


SAWYER'S FORCEPS.

Considerable discussion was had in the Association, touching the use of the forceps in obstetrics. Dr. Quackenbosh had read a paper before the New York State Society, that he had used the forceps fifteen hundred times. Dr. Newman, of Denver, advocated the use of the short forceps in the latter stages of labor, as a rule, and had devised a short forceps for that purpose. It was affirmed they might be used without the knowledge of the woman.(?) Sawyer's forceps is a modification of Newman's, and the best in existence. Many protested against the doctrine of the frequent use of the forceps.

